# Transcriptional Control of Apical-Basal Polarity Regulators

**DOI:** 10.3390/ijms222212340

**Published:** 2021-11-15

**Authors:** Katja Rust, Andreas Wodarz

**Affiliations:** 1 Department of Molecular Cell Physiology, Institute of Physiology and Pathophysiology, Philipps-University, 35037 Marburg, Germany; 2 Department of Molecular Cell Biology, Institute I for Anatomy, Faculty of Medicine and University Hospital Cologne, University of Cologne, Kerpener Str. 62, 50937 Cologne, Germany; 3 Cluster of Excellence—Cellular Stress Response in Aging-Associated Diseases (CECAD), University of Cologne, Joseph-Stelzmann-Str. 26, 50931 Cologne, Germany; 4 Center for Molecular Medicine Cologne, Faculty of Medicine and University Hospital Cologne, University of Cologne, Robert-Koch-Str. 21, 50931 Cologne, Germany

**Keywords:** cell polarity, gene expression, transcriptional regulation, Par complex, Scrib complex, Crumbs complex, epithelial to mesenchymal transition, asymmetric cell division

## Abstract

Cell polarity is essential for many functions of cells and tissues including the initial establishment and subsequent maintenance of epithelial tissues, asymmetric cell division, and morphogenetic movements. Cell polarity along the apical-basal axis is controlled by three protein complexes that interact with and co-regulate each other: The Par-, Crumbs-, and Scrib-complexes. The localization and activity of the components of these complexes is predominantly controlled by protein-protein interactions and protein phosphorylation status. Increasing evidence accumulates that, besides the regulation at the protein level, the precise expression control of polarity determinants contributes substantially to cell polarity regulation. Here we review how gene expression regulation influences processes that depend on the induction, maintenance, or abolishment of cell polarity with a special focus on epithelial to mesenchymal transition and asymmetric stem cell division. We conclude that gene expression control is an important and often neglected mechanism in the control of cell polarity.

## 1. Introduction

Cell polarity refers to the subcellular asymmetry of the plasma membrane, cytoskeleton, or cellular organelles and is vital for the function of a broad range of cell types [1,2,3,4,5,6,7,8,9]. Cell types that rely on cell polarity range from epithelial cells to asymmetrically dividing cells and include neurons, migrating cells, and zygotes. The regulation of cell polarity is of utmost importance for cellular function and cells employ a variety of mechanisms to ensure appropriate abundance and activity of polarity determinants. Localization and activity of polarity proteins is heavily regulated through kinases and phosphatases [10,11,12]. In addition, increasing evidence suggests that protein stability via proteasomal degradation contributes to polarity control [13]. While the regulation of cell polarity at the protein level is fairly well understood, it is less well understood how the expression of genes encoding polarity proteins is regulated. Research has identified a number of processes in which polarity genes are transcriptionally controlled [14,15]. Here we review the recent literature on how polarity gene expression coordinates cell polarity with particular focus on epithelial cells and asymmetrically dividing stem cells across species and identify common themes.

Epithelial cells exhibit apical-basal polarization with an outwards-facing apical membrane domain and a basal side that faces the extracellular matrix of the basal lamina. The lateral domains of epithelial cells are characterized by intercellular junctions that mediate cell-cell adhesion. These features are essential for normal tissue morphogenesis and function [16]. While the formation of epithelial tissues requires the establishment of cell polarity, the production of mesodermal tissues, through epithelial to mesenchymal transition (EMT), relies on the loss of cell polarity [17]. Furthermore, cell polarity is tightly connected to proliferation and cellular growth. Correspondingly, many cell polarity determinants have tumor suppressive or pro-oncogenic properties and have been reported to be mis-regulated in a variety of different tumors, particularly in those of epithelial origin [18,19].

Similar to how the mis-regulation of cell polarity in epithelial cells can promote tumorigenesis, the loss of cell polarity in asymmetrically dividing cells can have adverse consequences. During asymmetric cell division, cell polarity assumes a dual function in the orientation of the spindle apparatus as well as the localization of cell fate determinants along the division axis, which results in the subsequent asymmetric inheritance of cell fate determinants by the resulting daughter cells. Asymmetric cell division is a common motif in stem cell division and faulty cell polarity can result in stem cell loss or over-proliferation and confers a susceptibility to tumorigenesis [20,21].

The establishment and maintenance of cell polarity is particularly well characterized in epithelial tissues and the protein determinants involved are highly conserved among species (Table 1). 

**Figure 1:**
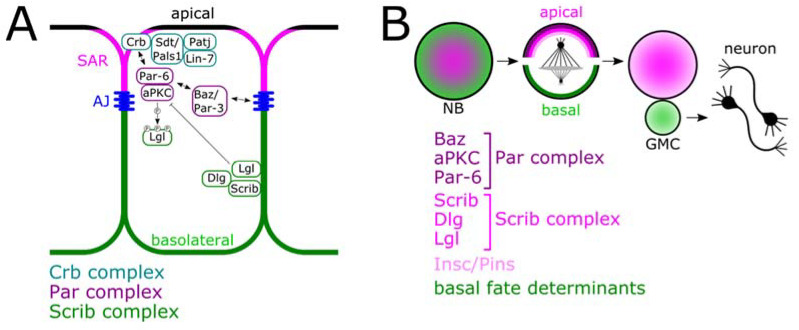
Overview of cell polarity determinants in *Drosophila* epithelia and neuroblasts. (**A**) Epithelial cells are polarized along the apicobasal axis. The Crb and Par complexes localize apically, and components of these complexes can interact with each other. In *Drosophila*, this region is referred to as the subapical region (SAR). In addition, Baz/Par-3 interacts with adherens junctions (AJ). The Par complex kinase aPKC phosphorylates Lgl, restricting the Scrib complex to the basolateral side of the cell, where it localizes cortically with the other members of the Scrib complex: Scrib and Dlg. At the basolateral side of the cell Lgl inhibits aPKC. (**B**) The *Drosophila* neuroblast (NB) is widely used as a model to study asymmetric stem cell division. Mitotic neuroblasts display apicobasal polarity. Components of the Par and Scrib complexes are apically localized. Further, Insc and its interactor Pins localize apically and orient the spindle apparatus. Neuroblast division results in two distinct daughter cells: the apical cell inherits apical determinants and maintains neuroblast fate. The basally formed cell, called ganglion mother cell (GMC), inherits basally localized differentiation factors and divides to produce neurons.

Epithelial polarity is controlled by three protein complexes that interact and cross-regulate each other (Figure 1A): the apically localized Partitioning defective (Par) and Crumbs (Crb) complexes, and the Scribble (Scrib) complex, which localizes basolaterally [24,25]. The Par complex consists of the atypical protein kinase C (aPKC), whose activity is controlled by its interactor Par-6 and the small GTPase cell division control protein 42 (Cdc42). Together with the scaffold proteins Par-3/Bazooka (Baz), Cdc42 also contributes to the localization of the aPKC-Par-6 complex [26]. The Par complex exerts a major function in regulating the phosphorylation status of polarity determinants, including components of the Par complex itself and of additional targets whose localization and/or activity is influenced by phosphorylation. The Scrib complex consists of the scaffold proteins Scribble (Scrib), Lethal giant larvae (Lgl), and Discs large (Dlg), all of which are composed of multiple protein-protein interaction domains. Lgl is excluded from the apical cortex through phosphorylation by aPKC. In turn, Lgl binds to the Par complex to inhibit aPKC, thereby rendering the kinase inactive in the basolateral cortex [27,28,29]. Like the Scrib complex, the Crb complex exerts its function via the regulation and facilitation of protein-protein interactions. The Crb complex contains the transmembrane protein Crb, which interacts with the scaffold protein Stardust (Sdt)/PALS1 via its intracellular domain, which in turn binds to PATJ and Lin-7. Members of the Crb complex can also form transient interactions with Par complex components [30]. For example, after aPKC-Par-6 are recruited via Baz/Par-3, the complex is handed over to Crb, which contributes to correct localization of aPKC-Par-6 [31].

Asymmetric stem cell division is often investigated using the *Drosophila* neuroblast model (Figure 1B). In contrast to their mutually exclusive localization in epithelia, the Par and Scrib complexes co-localize apically in neuroblasts, and phosphorylation of Dlg1 by aPKC disrupts Dlg1 autoinhibition, which then allows it to interact with the spindle orientation factor GukHolder [32]. In neuroblasts, the phosphatase PP2A dephosphorylates both Par-6 and Baz, which leads to reduced aPKC kinase activity [33,34]. However, the interaction of PP2A with the Par complex is not restricted to *Drosophila* neuroblasts. In epithelial cells PP2A also regulates aPKC, thereby functioning in a similar fashion [11,35].

Another important player in asymmetric cell division is the adaptor protein Inscuteable (Insc), which is required for apical localization of the Par complex and orients the spindle apparatus by interaction with the microtubule binding protein Mushroom body defective (Mud) and the adaptor protein Partner of Inscuteable (Pins) [26].

It is important to note that many polarity determinants also possess functions outside of polarity protein complexes. For example, outside of the Par complex, Baz interacts with the adherens junction (AJ) core component E-cadherin. In humans, two distinct Par-3 homologs function at AJs (PARD3B) and in the Par complex (PARD3) [36]. In addition, besides regulation of apical-basal polarity, aPKC is part of several additional signaling pathways and Scrib is an important regulator of planar cell polarity [37,38].

## 2. Polarity Gene Expression during Epithelial to Mesenchymal Transition

When epithelial cells become mesenchymal during EMT, the loss of cell polarity is a crucial prerequisite for this process [39]. Hence, the expression of polarity proteins has to be repressed permanently. Importantly, because EMT is one of the hallmarks of cancer progression, understanding the steps that result in EMT is of high clinical relevance [40].

The regulation of the cell adhesion molecule E-Cadherin is the most well characterized example of transcriptional regulation during EMT and has been reviewed in great detail in the past [23,41,42,43]. In short, the expression of the E-Cadherin encoding gene is directly regulated by a plethora of transcription factors (see Table 1), out of which the E-box binding factors SNAIL, SLUG, ZEB1, ZEB2, and Twist1/2 repress transcription and RUNX1, FOXA, p300, Rb, c-Myc, and AP-2 contribute to activation of transcription. Further, the chromatin regulators PRC2, G9a, and LSD1 as well as the Jak-Stat signaling pathway contribute to E-cadherin gene expression. During EMT, E-Cadherin downregulation is often accompanied by an upregulation of N-Cadherin, which is transcriptionally regulated by NFκB [44,45]. E-Cadherin and N-Cadherin expression is tightly linked. First, E-Cadherin represses NFκB via p38 MAPK, second NFκB induces the expression of the genes encoding the EMT-inducing transcription factors SNAIL, SLUG, ZEB2 and TWIST1 [45,46].

Several transcription factors regulating E-Cadherin transcription also target polarity protein expression and a few transcription factors that have not been described to regulate E-Cadherin transcription further influence EMT via the regulation of cell polarity (Table 1). Among these, the zinc finger transcription factor Snail has the biggest known repertoire of targets among polarity genes in different tissues and species [47]. Like its mammalian homolog, *Drosophila* Snail is competent to induce EMT-driven tumors, suggesting that studies conducted in model organisms are highly relevant [48]. Among the polarity regulators regulated by Snail, not all targets are directly regulated. For example, during *Drosophila* gastrulation, Snail represses Baz on the post-transcriptional level [49]. This Snail-mediated downregulation targets the junctional function of Baz, as it results in a decrease of E-cadherin at AJs without affecting other E-cadherin pools. Hence, this regulation of Baz contributes to the regulation of E-cadherin rather than the regulation of cell polarity complexes. The Snail-mediated repression of Baz is highly dynamic. During mesoderm internalization, when AJs shift apically in order to allow tissue folding, Baz repression is transiently blocked. While the authors did not test the exact mode of how the Snail represses Baz, several facts suggest that it may not take place at the level of transcription. First, *baz* mRNA is mainly maternally supplied during this stage of *Drosophila* embryogenesis. Second, when *baz* is expressed under the control of heterologous promoters, the protein is still removed from junctional sites and lastly, the regulation is highly dynamic in nature, further arguing for a regulation at the post-transcriptional level [49].

In contrast, the gene encoding human DLG1 is known to be directly bound by SNAIL. The regulatory region of the gene encoding this member of the Scrib complex contains several SNAIL consensus-binding E-box sites and is directly repressed by SNAIL during cancer progression of a variety of tumor types [50]. Thus, it is likely that SNAIL regulates *DLG1* during developmental EMT as well. Similarly, the human gene encoding the Lgl homolog LLGL2/Hugl-2 contains E-box sequences and is directly bound and repressed by SNAIL in breast cancer cells [51]. This repression of *LLGL2* is instrumental for SNAIL -mediated EMT, as the removal of the *LLGL2* E-box sites reverses the SNAIL -induced phenotype.

SNAIL does not only target the Scrib complex, but has a repressive effect on the Crb complex as well [52]. In Madin–Darby Canine Kidney (MDCK) cells, SNAIL represses transcription of the *CRB3* gene and also results in reduced transcription of the Crb complex components *PATJ* and *PALS1*. In contrast, Par and Scrib complex component levels were largely unaffected. The *CRB3* gene contains several E-box sequences, which are directly bound by SNAIL. Interestingly, SNAIL levels did not always correlate with the level of *CRB3* transcriptional downregulation, hence additional mechanisms must contribute to *CRB3* transcriptional regulation [52]. DamID experiments in *Drosophila* also revealed binding of Snail to the *crb* locus, consistent with *crb* downregulation during neuroblast selection, which is an EMT-like process [53].

E-box sequences can be bound by other transcription factors, including ZEB-1, which has been shown to repress *LLGL2*, *PATJ*, and *CRB3* in a breast cancer cell line [54]. Whiteman et al. [52] speculate that while SNAIL mediates the initial reduction of *CRB3* expression during EMT, other E-box binding transcription factors such as ZEB-1, ZEB-2, SLUG, and E47 are responsible for sustained *CRB3* repression. Another study conducted in breast cancer cells further supports the presence of SNAIL-independent regulatory mechanisms of *CRB3* expression. In this study, while SNAIL binding to the *CRB3* promoter was equally observed, this did not result in a relevant downregulation of *CRB3* transcript levels [55]. Instead, the transcription factor ZEB-1 associates with MUC1-C to directly repress *CRB3*. Furthermore, CRB3 is regulated by the transcription factor estrogen receptor α (ERα) in breast cancer cells. However, this regulation takes place post-transcriptionally and most likely occurs at the level of protein stability [56].

Human *CRB2*, but not *CRB3*, is repressed by the transcription factor hGATA6, named after its ability to bind to “GATA” DNA sequences. This regulatory mechanism was first discovered during *Drosophila* EMT, where the hGATA6 homolog Srp represses *crb* directly [57]. Srp also targets genes encoding other polarized proteins including the Crb complex member Sdt, the apically localized Stranded-at-second (Sas) and the basolateral Claudins Sinuous (Sinu), Megatrachea (Mega), and Kune-kune (Kune). However, the Par complex proteins Baz, aPKC and Par-6 are not affected by *Drosophila* Srp and the CRB interactors LIN-7 and PATJ are not targeted by human hGATA6 [57].

## 3. Polarity Gene Expression and the Regulation of Asymmetric Stem Cell Division

Asymmetric cell division allows stem cells to reproduce a stem cell, which inherits the stem cell specific factors, while differentiation factors are loaded into the second, differentiating the daughter cell. This mechanism promotes the rapid differentiation of the non-stem cell daughter and contributes to stem cell maintenance. In addition to the asymmetric inheritance of cell fate determinants, the continued expression of stem cell fate determinants has to be ensured in the mother cell. At the same time, it is sensible to employ mechanisms to repress the transcription of stem cell factors in differentiating daughter cells.

One such mechanism in which a stem cell factor is transcriptionally regulated during asymmetric stem cell division has been described in *Drosophila* neuroblasts. The Par complex kinase aPKC promotes neuroblast self-renewal and is apically localized during division and thus inherited by the neuroblast daughter. In the neuroblast, aPKC participates in a feedback mechanism resulting in its transcriptional regulation [58]. aPKC phosphorylates the transcription factor Zinc-finger protein (Zif), which prevents Zif nuclear entry. Upon neuroblast division, the basally formed ganglion mother cell accumulates unphosphorylated Zif, which enters the nucleus to directly repress *aPKC* transcription. This promotes differentiation and blocks reversion to a stem cell-like state. Interestingly, neuroblasts display nuclear Zif localization despite the aPKC-mediated nuclear exclusion of Zif, suggesting that not the entire Zif protein pool is phosphorylated in neuroblasts. In agreement, *zif* mutation does not only lead to a failure in daughter cell differentiation but also results in phenotypes in the neuroblast itself: *zif* mutant neuroblasts display mislocalization of polarity determinants, which is mostly rescued in an *aPKC* heterozygous mutant background. Hence, while Zif functions to repress *aPKC* in ganglion mother cells to allow differentiation, it fine tunes *aPKC* levels in neuroblasts to regulate cell polarity [58].

In the larval neuroblast, the continued expression of *aPKC* is ensured by the transcription factor Myc, which is well known for its roles in cell cycle progression and positive regulation of cell growth [59]. Myc binds to the *aPKC* gene and recruits the Tip60 chromatin remodeler complex, which increases the permissive euchromatin marks H4K8Ac and H2Av and induces *aPKC* expression. Knockdown of components of the Myc-Tip60 complex or *aPKC* led to loss of apical-basal polarity. Neuroblasts were smaller and divided symmetrically and were ultimately lost by premature differentiation via nuclear entry of the aPKC target and transcription factor Prospero. Interestingly, restoration of aPKC levels restored apical-basal polarity in Myc-Tip60 complex knockdown but failed to rescue asymmetric division and Prospero nuclear entry. The persistence of symmetric divisions in Myc-Tip60 knockdown rescued with *aPKC* overexpression can be traced to several Myc-Tip60 targets regulating the spindle and centrosomes while the premature nuclear entry of Prospero is likely connected to the impact of Myc-Tip60 on cell size. Together, Myc and the Tip60 complex regulate not only apical-basal polarity in neuroblasts but are vital for neuroblast growth and asymmetric division [59].

The interaction between Myc and the Tip60 complex is conserved in human embryonic stem cells (ESCs) [60,61] and polarity genes are expressed in these cells, although their role is poorly understood [62,63]. Interestingly, MYC controls the balance between symmetric and asymmetric cell division in human neuroblastoma, further supporting a conserved function in cell polarity control [64].

When differentiation towards the mesendoderm is induced in mouse ESCs, these stem cells switch between symmetric and asymmetric division to balance self-renewal and differentiation. Asymmetric division is regulated by INSC, which interacts with PAR-3 and LGN/PINS. INSC orients the spindle apparatus, similar to the function of Insc in *Drosophila* neuroblasts. INSC levels determine whether ESCs divide symmetrically or asymmetrically, whereby high INSC levels induce asymmetric division. Hereby, the NF-κB-familiy transcription factor reticuloendotheliosis oncogene (c-Rel) binds to the *INSC* promoter and induces *INSC* transcription. This leads to increased rates of asymmetric division, which ultimately promotes mesodermal cell fates [65].

In *Drosophila* embryonic neuroblasts, the Snail-family transcription factors Escargot (Esg), Snail and Worniu (Wor) indirectly regulate *insc* expression [66,67]. These studies depict a prime example illustrating how vital it is to consider potential indirect mechanisms when studying transcription factors: Careful dissection into the mechanism revealed that both transcription as well as translation of *insc* are indirectly regulated by the transcription factor triad. First, Snail binding to its co-repressor C-terminal binding protein (CtBP) is crucial for Snail-mediated neuroblast specification. Yet, *insc* transcript levels are positively regulated by Snail, Esg, and Wor and hence *insc* transcription is likely indirectly induced [67]. While Esg, Snail, and Wor regulate *insc* transcription during early stages of neurogenesis, *insc* transcription is further regulated by an unknown additional mechanism during the later stages, as *insc* mRNA can be detected in an *esg, snail*, *wor* triple mutant in a delayed manner [66]. However, this transcriptional induction is insufficient to restore Insc protein levels in the *esg, snail*, *wor* mutant background. Thus, in addition to the transcriptional regulation, Esg, Snail, and Wor regulate *insc* translation, which requires the 5′ and/or 3′-UTRs of the *insc* mRNA. The nuclear localized Esg, Snail, and Wor proteins are unlikely to directly cause this translational regulation and instead most likely regulate *insc* translation via other genes. Thus, both the transcriptional as well as translational regulation of *insc* by Esg, Snail, and Wor are indirectly mediated. Moreover, while *esg, snail*, *wor* triple mutant neuroblasts display completely randomized spindle orientation, *insc* deficient neuroblasts display normal spindle positioning during telophase [66]. This so-called “telophase rescue” depends on Dlg1 and Kinesin heavy chain 73 (Khc73) [66,68,69]. The absence of this telophase rescue in *dlg* and *khc73* mutants suggests that Dlg1 and/or Khc73 could be targets of these Snail family transcription factors in neuroblasts.

## 4. Polarity Gene Expression in Other Processes

Besides EMT and asymmetric stem cell division, several other processes require a fine regulation of cell polarity. These include establishment of cell-cell contacts, cell cycle progression, and differentiation [70,71]. In humans, *DLG1* is one of the genes encoding polarity proteins targeted for expression control during a number of these processes. Depending on the process, *DLG1* expression is regulated at the transcriptional level alone or in combination with translational efficiency via the expression of splice variants. Alternative splicing of *DLG1* mRNA results in either a large or a short isoform, which encode for the same protein but differ in the 5′UTR [71]. The longer isoform is translated with lower efficiency than the shorter isoform, which is likely due to more stable secondary RNA structures of the longer isoform. Depending on the required levels of DLG1 protein, the long and short isoforms are expressed in specific ratios that allow for the fine tuning of DLG1 protein levels [70]. The factors regulating transcription and alternative splicing of *DLG1* mRNA are unknown, with the exception of repression through the transcription factor Snail during EMT, as mentioned above [50].

Studies conducted in several model organisms provide further insight into the mechanisms of transcriptional control of Dlg. During *C. elegans* epithelium formation, *dlg-1* transcription is induced by PHA-4 [72]. PHA-4 acts as a pioneer transcription factor and also induces the expression of other epithelial genes including *par-3*. In contrast, *par-6* mRNA is maternally deposited and the zygotic transcriptional control of *par-6* does not play a role during epithelial formation in *C. elegans* [72,73,74,75]. Upon transcription of *dlg-1*, the kinesin ZEN-4 interacts with its binding partner CYK-4 to regulate DLG-1 protein accumulation, as well as the accumulation of other polarity proteins including PAR-6, PKC-3/aPKC, and PAR-3 [72,76]. Whether ZEN-4 and CYK-4 regulate translation or protein stability of these cell polarity determinants is currently unknown.

During the development of the *Drosophila* wing disc epithelium, *dlg1* is transcriptionally regulated. Wing disc development is regulated by the Dpp signaling pathway, which in turn induces the transcription of the Zinc finger transcription factors Spalt major (Salm) and Spalt-related (Salr). Salm and Salr appear to activate the expression of Dlg1 as well as its interactor Scrib [77]. In *Drosophila* neurons, it is clear that translational regulation contributes to Dlg1 protein expression levels. Here, the mRNA-binding protein Syncrip regulates Dlg1 protein levels and localized mRNA translation in order to regulate synaptic growth [78]. Besides, *Par-3*, *sdt*, and *crb* mRNAs have been shown to display distinct subcellular localizations in neurons (*Par-3*) or epithelial cells (*sdt*, *crb*), suggesting that local translation contributes to the regulation of cell polarity [79,80,81].

The expression of several Par complex members was shown to be regulated in a variety of tissues. In the developing *Drosophila* wing, aPKC protein levels are increased via Hedgehog signaling in a positive feedback loop [82]. Together with Par-6, aPKC contributes to the activation of Hedgehog signaling via the phosphorylation of Smoothened. In turn, aPKC protein levels increase in a cubitus interruptus (ci) dependent manner. Whether or not aPKC expression is regulated at the transcriptional or post-transcriptional level and whether this is a direct regulation or a secondary effect is unclear, but since ci encodes a transcription factor, a direct transcriptional regulation is conceivable. Importantly, besides Par-6, no other polarity determinants contribute to aPKC-mediated Hedgehog activation, suggesting that the regulation of Hedgehog signaling is not directly linked to cell polarity. Significantly, the role of aPKC in Hedgehog signaling activation is conserved in mammals [83] and studies in avian species show that Hedgehog signaling regulates cell polarity during neural tube formation [84]. A post-transcriptional mode of ci-mediated regulation of aPKC might represent a possibility for a targeted regulation of aPKC functions in Hedgehog signaling. Yet, transcriptional regulation of aPKC may contribute to the regulation of both the cell polarity- as well as the Hedgehog-mediating functions of aPKC.

In the *Drosophila* eye, aPKC inhibits the planar cell polarity (PCP) pathway via the phosphorylation of Frizzled1 (Fz1) upon its recruitment by Patj [85]. In turn, PCP signaling results in the downregulation of aPKC and Patj protein levels, while Baz protein levels are upregulated. Baz has been shown to repress aPKC kinase activity and, consistently, represses aPKC-mediated phosphorylation of Fz1. Presently, it is unclear whether the differential regulation of these polarity determinants occurs at the transcriptional or post-transcriptional level.

During heart development, a mechanism that is conserved from *Drosophila* to mammals, aids in the regulation of *cdc42*. The transcription factor Tinman in *Drosophila* or its mouse homolog Nkx2-5 positively regulate *cdc42* levels. In mammals, Nkx2-5 represses the micro-RNA miR-1, which in turn negatively regulates *CDC42* [86]. While the function of the Par complex in mammalian cardiomyocytes has not been determined yet and CDC42 has several functions outside of polarity regulation, CDC42 is important for cell-cell adhesion during heart development [87]. This suggests that the Tinman/Nkx2-5 mediated regulation of *cdc42* could contribute to the regulation of Par complex function.

*Drosophila* spiracle development requires extensive cell shape changes that are associated with the differential regulation of cell polarity regulators as well as cytoskeletal changes [88,89]. Spiracle development is induced by the Hox transcription factor Abd-B, which induces the expression the genes encoding the transcription factor Cut and the Jak-Stat ligand Upd. In turn, the Jak-Stat effector Stat92E directly induces *crb* expression. However, restoration of Crb levels in a Jak-Stat deficient background cannot rescue the Jak-Stat induced phenotype. This might be due to other crucial targets of Stat92E in spiracle development such as *shg*, encoding *Drosophila* E-Cadherin, which is further controlled through Cut. Here, it should be noted that it was not tested whether the *shg* gene is indeed bound by Cut or Stat92E or if either could regulate *shg* expression indirectly [88].

While the phosphatase PP2A is not a member of the Par complex, it is important for regulating its activity [33,34,35,90]. PP2A consists of the scaffold subunit A, which interacts with Baz in *Drosophila* [33], a regulatory B subunit, and the catalytic C subunit, which confers the serine/threonine phosphatase activity. In mammals, several versions of each subunit exist that are encoded by distinct genes (Table 1). Four families of B subunit versions dictate target specificity. The A and C subunits each are encoded by an A and a B gene. The genes *PPP2CA* and *PPP2CB* encoding the scaffold subunit PP2A-A are differentially regulated, with a manifold higher expression of the *PPP2CA* gene on both the transcript as well as the protein levels. Mis-regulation of PP2A-A has been implicated in several diseases [91]. Correspondingly, the expression of both *PPP2CA* and *PPP2CB* genes have been reported to be tightly regulated by a variety of transcription factors. The *PPP2CA* gene is positively regulated by the transcription factors CREB, ETS-1 and AP-2α and negatively regulated by SP-1, all of which have been reported to directly bind to the *PPP2CA* promoter [92]. The *PPP2CB* gene is positively regulated by SP1/SP3 and RXRα/β and negatively regulated by ETS-1 through direct interaction [93]. The functional implications of these extensive regulatory mechanisms have not been described and PP2A targets many proteins outside of the Par complex. For example, it regulates a number of junctional proteins and plays an important role in cell cycle regulation [11,94]. Yet, these transcription factors should be considered candidates for potential regulators of cell polarity determinants.

## 5. Discussion

Among the various processes requiring differential regulation of cell polarity determinants, the regulation of EMT is particularly well understood. Strikingly, the transcription factors Snail and ZEB-1 share a common target motif—the E-box sequence “CANNTG”. Further, the “GATA” sequence is a motif regulated during EMT across species. While both these target sequences display low complexity, and thus sequence analysis alone is insufficient to predict potential new targets, these motifs should be considered in more detail by studies investigating the transcriptional control of cell polarity determinants.

The E-box binding transcription factor SNAIL is known to target a variety of cell polarity determinants during EMT. The genes encoding DLG1, LLGL2, and CRB3 contain confirmed E-boxes and are directly bound and repressed by SNAIL [50,51,52]. Snail further regulates Baz, Patj and Pals1 [49,52]. While it is unclear whether Patj and Pals1 are directly regulated, Baz is unlikely to be targeted directly. A different study found that the Snail-family transcription factors Snail, Esg, and Wor did not target Baz at the transcriptional level in neuroblasts [66]. Thus, Baz is unlikely to be a direct target of Snail in *Drosophila* epithelia and neuroblasts.

Together with Esg and Wor, Snail has an important function in the regulation of cell polarity during asymmetric neuroblast division. This triad of transcription factors indirectly activates *insc* transcription and translation [66,67]. Further, Snail, Esg, and Wor appear to target key factors of the telophase rescue program [66]. Dlg1, one of the components inducing telophase rescue in flies [68,69], is directly repressed by Snail during EMT in a number of different tumors in mammals [50]. In MDCK cells, DLG is mildly downregulated by SNAIL, although it is unclear whether this is an effect of direct SNAIL binding [52]. However, the requirement for Snail, Esg, and Wor for telophase rescue in *Drosophila* neuroblasts rather points to a positive effect on cell fate determinants such as Dlg1. Hence, the transcriptional repression of *dlg1* by Snail is not conserved in neuroblasts and may not be of major relevance in mammalian epithelia either.

In contrast, the *crb* promoter binding by Snail is conserved between mammals and flies [48,52]. Interestingly, while this binding leads to repression of transcription in MDCK cells, it does not constitutively result in *CRB3* repression in breast cancer cells [52,55].

Together, Snail indisputably targets cell polarity determinants in order to regulate EMT. However, which cell polarity determinant is targeted depends on the cell type and organism. While some Snail targets may simply not be conserved, the regulation of *crb* depicts an example that suggests that Snail targets are context-dependent. It will be interesting to learn about which cofactors regulate the exact choice of Snail target genes.

It is unclear to what degree other mechanisms regulating the expression of polarity determinants are conserved. For example, the FoxA family transcription factor PHA-4 induces polarity determinant expression in the worm [72]. While *C. elegans* PHA-4 is a driver of organogenesis, the *Drosophila* PHA-4 homolog Forkhead rather regulates cellular function than cell fate specification [95]. The role of the Forkhead family transcription factors in the induction of polarity determinant expression may therefore not be conserved.

The regulation of the genes encoding the PP2A subunit A is influenced by several transcription factors but the functional implications of these regulatory mechanisms are unclear [92,93]. However, PP2A has been assigned a variety of functions independent of its function in cell polarity [91,94]. Whether one of the transcription factors regulating PP2A-A expression influences cell polarity is unclear. Yet, among the PP2A-A regulating transcription factors, ETS-1 may represent a potentially interesting candidate as it was shown to regulate endothelial cell-matrix adhesion [96].

An intriguing commonality between the examples described above is the fact that in most cases a single or only a few cell polarity determinants are transcriptionally regulated. This is the case during EMT where *DLG1* and *CRB3* are frequent targets and during asymmetric division where *aPKC* and *insc* are common targets. It remains to be investigated why some transcriptional control contributes to the regulation of some polarity determinants more than others. A potential explanation could be that misexpression of transcriptionally regulated polarity determinants is highly unfavorable. For example, *dlg1* mutation facilitates tumor development and aPKC and Insc are crucial self-renewal factors during asymmetric stem cell division [20,97]. Otherwise, it might be more energy and/or time efficient for cells to regulate one or only a few key regulators of polarity. Through interactions at the protein level, these key regulators could then organize changes in the polarity system. In addition, in many cases we may simply not yet know of the transcriptional regulation of polarity regulators.

## 6. Conclusions and Remarks

Many processes require the fine tuning of cell polarity. How polarity regulators are regulated on the protein level is extensively studied and has revealed that many mechanisms regulate polarity regulator activity, stability, localization, and translation. Beyond this, through our literature review we further conclude that transcriptional regulation is decisive for many processes where cell polarity needs to be established, maintained, or abolished. While some transcriptional regulation mechanisms have been described, much remains to be learned about transcriptional regulation of polarity determinants. A particularly interesting task constitutes the investigation of why some polarity determinants are transcriptionally regulated while others are not and how target choices of transcription factors are made based on the cellular context.

## Figures and Tables

**Table 1:** Apicobasal polarity proteins are conserved across species.

Human	*D. melanogaster*	*C. elegans*	Upstream Transcription Factors
	Par complex		
*PARD3*	*baz*	*par-3*	Snail (*Drosophila*, gastrulation) **PHA-4** (*C. elegans*, epithelium)
*PARD6A*	*par-6*	*par-6*	
*PKCλ, PKCζ*	*aPKC*	*pkc-3*	**Zif** (*Drosophila*, neuroblast) **Myc-Tip60 **(*Drosophila*, neuroblast) ci (*Drosophila*, wing)
*CDC42*	*cdc42*	*cdc-42*	Tinman/Nkx2-5 (*Drosophila*/mouse, heart development)
	Scrib complex		
SCRIB *	*scrib*	*let-413*	**Salm** and **Salr **(*Drosophila*, wing)
*DLG1, DLG2, DLG3, DLG4*	*dlg1*	*dlg-1*	**Snail **(human, tumorigenesis) **PHA-4** (*C. elegans*, epithelium) **Salm** and **Salr **(*Drosophila*, wing)
*LLGL1, LLGL2*	*l(2)gl*	*lgl-1*	**Snail** (human, breast cancer) **ZEB-1** (human, breast cancer)
	Crumbs complex		
*CRB1, CRB2, CRB3*	*crb*	*crb-1, crb-3*	**Snail **(MDCK) **ZEB-1** (human, breast cancer) **MUC1-C** (human, breast cancer) ERα (human, breast cancer) **hGATA6/Srp **(human/*Drosophila*, EMT) **Stat92E **(*Drosophila*, spiracle)
*PALS1*	*sdt*	*magu-2*	Snail (MDCK) Srp (*Drosophila*, EMT)
*PATJ*	*patj*	*mpz-1*	Snail (MDCK) **ZEB-1** (human, breast cancer)
*LIN7A, LIN7B, LIN7C*	*veli*	*lin-7*	
	Selected interactors of polarity complexes		
*INSC*	*insc*	*insc-1*	**c-Rel **(mouse, ESC) Escargot and Snail and Worniu (*Drosophila*, neuroblast)
*CDH1*	*shg*	*hmr-1*	**SNAIL, SLUG, ZEB-1, ZEB-2, Twist1/2, RUNX1, FOXA, p300, Rb, c-Myc, AP-2** (recently reviewed in [22]) ct, Stat92E (*Drosophila*, spiracle)
*CDH2*	*CadN*	*hmr-1*	**NFκB**
*PPP2R2/3/5/6* *PPP2CA/B* *PPP2R1A/B*	*Pp2A-29B, mts, wrd*	*let-92, paa-1, sur-6, pptr-1/2, rsa-1, cash-1*	**CREB**, **ETS-1, AP-2α,** **SP-1, SP-3, RXRα/β ** **(**mammal, epithelium**)**

The table lists genes encoding cell polarity determinants and their orthologs in human, *Drosophila melanogaster,* and *Caenorhabditis elegans*. Upstream regulators are **bold** when they are known to directly affect transcription of their targets, underlined when they affect their target indirectly and in normal letters when the exact mechanism is unclear. Magenta text indicates repression, green text indicates activation of gene expression. * Two related proteins, ERBIN (enoded by *ERBIN*) and LANO (encoded by *LRRC1*), act redundantly with SCRIB in mammals [23]. MDCK: Madin-Darby Canine Kidney cells.
